# Recent Advances in Polyurethane/POSS Hybrids for Biomedical Applications

**DOI:** 10.3390/molecules27010040

**Published:** 2021-12-22

**Authors:** Jan Ozimek, Krzysztof Pielichowski

**Affiliations:** Department of Chemistry and Technology of Polymers, Cracow University of Technology, Warszawska 24, 31-155 Cracow, Poland; kpielich@pk.edu.pl

**Keywords:** polyurethane, POSS, functionalization, hybrids, biomedical applications

## Abstract

Advanced organic-inorganic materials-composites, nanocomposites, and hybrids with various compositions offer unique properties required for biomedical applications. One of the most promising inorganic (nano)additives are polyhedral oligomeric silsesquioxanes (POSS); their biocompatibility, non-toxicity, and phase separation ability that modifies the material porosity are fundamental properties required in modern biomedical applications. When incorporated, chemically or physically, into polyurethane matrices, they substantially change polymer properties, including mechanical properties, surface characteristics, and bioactivity. Hence, this review is dedicated to POSS-PU composites that have recently been developed for applications in the biomedical field. First, different modes of POSS incorporation into PU structure have been presented, then recent developments of PU/POSS hybrids as bio-active composites for scaffolds, cardiovascular stents, valves, and membranes, as well as in bio-imaging and cancer treatment, have been described. Finally, characterization and methods of modification routes of polyurethane-based materials with silsesquioxanes were presented.

## 1. Introduction

The aging population and increase in living standards has caused an increased need for biomaterials, which have seen rapid growth in recent decades [1]. The resulting significant technological progress [2] has led to third-generation biomaterials, the biomimetic systems that imitate nature-derived solutions found in the living organism [3]. This revolution was possible due to multidisciplinary collaborations between chemists, physicists, biologists, material scientists, engineers, and surgeons [4]. By applying different materials, such as polymers (e.g., polyurethanes, silicones, and acrylic polymers) and biopolymers (e.g., collagen, polysaccharides, polyhydroxyalkanoates), metals and alloys, ceramics and glasses—e.g., hydroxyapatite, silica, and alumina, and the combination thereof—it was possible to develop new composite and hybrid materials with superior properties. However, since the behavior of surrounding tissue and body environment decides the acceptance or rejection of a biomaterial, governed by its physical and chemical properties of the surface, and mechanical properties, finding the material possessing proper properties is a challenging task [5]. Therefore, research efforts focus on the advanced materials-composites, nanocomposites, and hybrids having different contents of organic and inorganic components, to achieve properties required for biomedical applications [6].

Among those materials, nanocomposites have at least one dimension of the components ranging up to 100 nm. Such small sizes of nanostructures result in uncommon features of those materials because of prevailing surface properties over the material’s bulk properties. There are two main strategies for obtaining such materials: top-down and bottom-up. In the first case, the bulk material is split up to form nanomaterial, while in the second strategy, the nanomaterials are assembled from building blocks. Those building blocks, like molecules or clusters, are obtained from the bottom: grown from the elemental precursors. Nanostructures obtained by these techniques seem to be a key in producing nature mimicking biomaterials since the natural structures are hierarchical. In this field, the polymeric nanocomposites that contain inorganic nanofillers combine beneficial features of both phases, and even synergistic effects may be observed. For example, adding a nanofiller may result in better thermal and chemical resistance, mechanical properties (strength, modulus, hardness), as well as electrical and magnetic properties [7].

On the other hand, the polymer matrix provides processability, lightweight, and flexibility. The key to this approach’s success is to keep a good interaction between nano and polymeric phases resulting in good homogeneity of a dispersed phase in the continuous phase. Therefore, there is tremendous interest in nanocomposites in which covalent bonds are formed between the phases, resulting in homogeneity and stability of the material, e.g., migration of the filler is prohibited. On the other hand, covalent bonding of the phases with low compatibility results in phase-separated, self-assembling materials showing biocompatible and biostable properties [8]. When organic and inorganic molecules form a covalent bond in-between, the resulting material is called a hybrid (organic-inorganic) biomaterial.

One class of the finest well-defined nanoparticles are polyhedral oligomeric silsesquioxanes (POSS), considered the tiniest glass nanoparticles. Although they were first synthesized in the 1870s, they are a new, rediscovered generation of zero-dimensional nanomaterials, with the structure resembling a cage in which corners are silicon atoms bound with oxygen bridges [9]. Their empirical formula is (RSiO1.5)n, in which R can belong to the great variety of chemical substituents, while n = 6, 8, 10, 12, 14, 16, 18. Because POSS represent a large group of compounds, the potential biomaterial structures with resulting tailored properties and well-defined structures are almost unlimited [10]. These fascinating molecules located between silica and polysiloxanes combine benefits of both groups being thermally, chemically, and radiation stable, with ease of processing and manipulation using conventional chemical techniques. Moreover, their properties (chemical and physical, like solubility) are tailorable by the composition of R substituents [7]. POSS cages may be unreactively dissolved in the polymer matrix or reacted as a pendant or bead-like comonomer, also used as a cross-linker, grafting molecule, or net nodes. When added into the polymer matrix, they remain non-toxic, improve mechanical properties, and resist biodegradation, which is very important in tissue engineering. These properties result in broad medical applications of such materials [11], e.g., components in hard-tissue biomaterials [12,13], scaffolds [14,15,16,17], fibers [18,19], drug delivery systems [20,21,22], dendrimers [23,24], photodynamic therapy [24,25], and medical imaging techniques [7,24,26]. POSS is a key component in dental materials, improving mechanical properties and extending the service life [7]. Recently, methacrylate octa-armed silsesquioxanes were synthesized with high yields and reacted with ethylene glycol dimethacrylate (EGDMA) and 2-hydroxyethyl methacrylate (HEMA) to form three-dimensional scaffolds for hard tissue replacement [12]. In the case of brand new POSS employment in medicine, a material with POSS interconnected by pyrene is worth mentioning since application in fluorescent detection and removal of antibiotics is a completely new branch of POSS utilization [26].

Polyurethanes (PUs) are a group of polymers that possess urethane bonds in the main chain. POSS has been used in polyurethanes for multiple applications. Noteworthy, POSS-PU nanocomposites are known to have higher thermal stability [27,28,29,30,31], especially when nonreactive substituents are phenyl groups. Those kinds of POSS most efficiently promote the formation of silica-containing char that inhibits further degradation [32]. Less stable kinds of POSS, with isobutyl substituents, appear to be more stable in copolymerized form and enhance the thermal stability by reduction of chain mobility and blockage of some paths of degradation; however, the presence of POSS attached in hard segments may distort more thermally stable hard domains [33,34,35,36]. Besides thermal properties, POSS presence in polyurethanes influences, e.g., the tensile strength [29,37,38] and reduces the dielectric constant [37,39,40].

In medicine, PU are important materials since they can be simultaneously elastic and thermoplastic (thermoplastic polyurethane–TPU) or porous (rigid and flexible foams); by choosing the amount and type from a variety of polyols and isocyanates, they have the potential of tailorable properties. Moreover, they show good blood compatibility and may be a biostable and biodegradable component in medical applications due to a wealth of possible compositions. The ease of properties tailoring is crucial as the material has to fit the properties of surrounding tissue. For instance, PU biomaterials in the form of foams can replace soft tissues. However, as they are traditionally obtained from the most reactive aromatic isocyanates, they are believed to leak carcinogenic 2,4-toluenediamine (TDA) [41,42]. Even though the FDA approved polyurethane-coated breast implants as a material of low risk for health, it is also possible to omit aromatic isocyanates and produce aliphatic PU more safely [43,44].

Hence, the exciting idea in this field is to produce non-isocyanate polyurethane (NIPU) obtained most promisingly from cyclic carbonates reacting with amines [45,46,47]. During the opening of the ring of cyclic carbonate, either primary or secondary hydroxyl group emerges. The presence of these groups results in high hydrophilicity of those materials, which is a desirable property in the case of biodegradable materials. Moreover, the use of epoxidized unsaturated oils in cyclic carbonates production gives a potential to obtain biomaterial from renewable resources that should be easily resorbable by the living tissue [48]. NIPU materials develop rapidly, and there are already some reports on their progress, e.g., through electrospinning to fabricate mats for biomedical applications [49]. They were also proven to have antibacterial properties when a star-shaped macromonomer was used to produce a surface coating [50].

This review is dedicated to POSS-PU composites and comprises four major parts. In the Section 2, we discuss the possible ways of POSS incorporation into the polyurethane matrix. In the Section 3, we review the described literature findings from the application point of view. Finally, in the Section 4, the reader may find the main ways of characterization of those materials, while in the Section 5, the methods of obtaining and modifying this kind of hybrid material are described.

## 2. Methods of PU/POSS Synthesis

**Figure 1:**
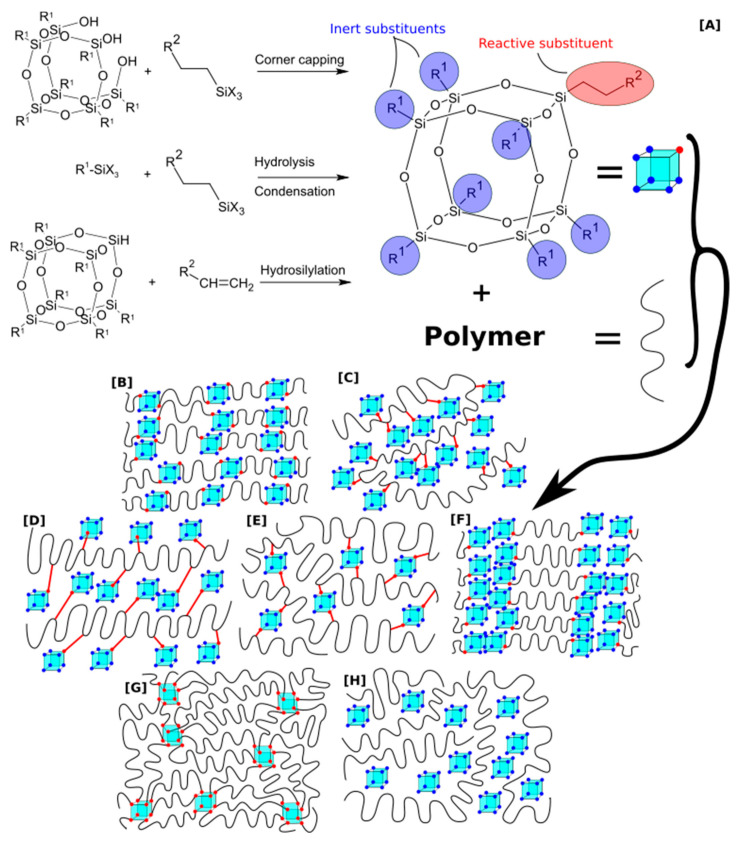
The scheme of POSS synthesis and incorporation into polymer matrices. (**A**) Most popular reactions, (**B**–**H**) various ways of incorporation of POSS, (**B**) pendant, (**C**) crosslinker, (**D**) bead-like, (**E**) physical blend, (**F**) telomeric, (**G**) net nodes, (**H**) grafted.

In POSS molecules, functionalization is possible at the silicon corners of the cage. For example, the most commonly used cube-shaped T8 system contains eight silicon corners to which any organic group can be attached. Functionalization is possible at the stage of molecule formation or later through the hydrosilylation reaction. In the case of polyurethanes, functional groups enabling dissolution in the matrix are often used, e.g., tert-butyl, phenyl, cyclohexane, and various types of functional groups, such as isocyanates, amines, epoxides, and alcohols. POSS can be introduced in various ways into the system, which affects the macromolecular architecture and the degree of homogenization. For example, POSS molecules can be grafted on macromolecules, introduced as pendant groups, telomerised, cross-linked, and arranged in nets (Figure 1).

### 2.1. Copolymerization

#### 2.1.1. Rigidly Bonded

Rigidly bonded POSS are usually those with partially condensated cages and functional groups attached to both sides of the cage (Figure 1B) POSS in the form of rigidly incorporated comonomer can be a bifunctional compound with a partially condensed cage having silanol groups [51,52,53,54,55] or having functional groups on both sides of the cage [56,57,58]. The first studies were conducted in the US Air Force Research Laboratory [59,60,61]. POSS bearing two hydroxyl groups, and functionalized with bisphenol A, was incorporated into rigid segments of a polyurethane consisting of MDI and PTMG with a 2000 g/mol molar mass. In other studies, the influence of BPA-POSS on the reinforcement of rigid segments was examined [56,58]. POSS was introduced as a chain extender in these applications, and structural investigations confirm the chemical incorporation of silsesquioxane into the polymer chain.

The open-cage POSS is used in another approach in which a reaction between isocyanate and silanol groups occurs. For instance, three-functional open cage POSS (trisilanol) was used to react with HDI; DBTL was applied as a catalyst [51]. Trisilanol POSS was also used as a comonomer to produce hybrid poly(dimethylsiloxane-urethane) POSS showing low permeation of O_2_/N_2_ and CO_2_/N_2_ gasses [62].

An exciting class of silsesquioxanes are double-decker silsesquioxanes (DDSQs) that possess two reactive groups on each side of the molecule and two siloxane “decks” parallel to them. Recently, DDSQ with two hydroxyl groups on both sides has been reacted with HDI and PCL to form a hybrid material characterized by increased thermal stability, surface hydrophobicity, and lipophobicity [63,64,65].

#### 2.1.2. Pendant

POSS can be attached as pendant groups on the polymer chains by using a difunctional POSS substituted with one chain containing two reactive functional groups [43,66,67,68] (Figure 1C). These reactive groups can form a bond with isocyanates and build into the backbone while POSS hangs perpendicularly on the sides. Such an approach with POSS containing two hydroxyl groups on a flexible chain with an ether bond (PHI-POSS) was presented in the literature [69,70], where MDI, PTMG with a mass of 1400 g/mol, and BDO as a chain extender were used. The same PHI-POSS was found to change the thermal degradation mechanism in aliphatic PUs synthesized with HDI, PTMG, and BDO [34].

### 2.2. Chain Modification

#### 2.2.1. Grafting

Grafting is a method in which a previously obtained polymeric chain or surface is grafted with POSS, usually to add functionalization or increase compatibility when blending with another polymer [71] (Figure 1D). For example, the functionalization with POSS was done on carbon fibers previously modified by amino groups to obtain better interactions between the fibers resulting in greater tensile strength [72]. In addition, the grafting approach may lead to better homogeneity of the POSS dispersion [73], reduction of the polymer crystallinity [74], and formation of a protective layer against, e.g., hydrolysis [75].

#### 2.2.2. Crosslinking

Crosslinking is a network formation process, and POSS with multiple functionalities can act as efficient crosslinking agents (Figure 1E). Hence, a trisilanol-POSS was used in the reaction with HDI and PDMS to yield a prepolymer which was then cross-linked with the eight-functional octakis(hydroxydimethylsiloxy) POSS [62]. The amount of POSS crosslinker strongly influences the obtained material’s optical, electrical, and morphological properties [76]. It was found that POSS crosslinking may disorder hydrogen bonding in hydrogels, which causes lowering Tg value and increasing Young’s modulus [27]. 

#### 2.2.3. Telomerization

Chain termination/telomerization with POSS facilitates phase separation effects resulting in improved hydrophobicity and contact angle increase [77] (Figure 1F). Moreover, it changes morphology toward biocompatibility enhancements [29,30,31,32,33,34,78]. Notably, POSS can hold a single-NCO group [36,37,38] or hydroxyl [6] substituents in telomerised polyurethanes, and those functional groups can be further utilized.

#### 2.2.4. Net Nodes

The high functionality of POSS can be used to create polymer networks, in which POSS cages act as enhancements (Figure 1G). POSS is most often used as a polyfunctional compound with multiple (preferentially eight) isocyanate [29,51,79], hydroxyl [67,80,81], or amino [82,83] functional groups. Liu et al. prepared a polymer network using TDI, PPG (molar mass 1000 g/mol), MOCA as a chain extender, and Ope-POSS containing eight propylglycidyl groups on the flexible ether chain [81]. POSS content ranged from 5, 10, 15, and 20 wt%. The polymer network was also obtained in other POSS architectures, containing 10 and 12 silicon atoms (T10, T12), and used in contents from 0 to 52%. The network was synthesized in DMAC as a solvent, while IPDI and PTMG (molar mass 1000 g/mol), were used as an isocyanate component and a macrodiol, respectively. As a result, homogeneous and transparent products were obtained [83].

In addition to bulk polymerization, solvent polymerization is of interest, where POSS can be introduced in a solution, facilitating its homogenization. Since copolymerization is completed, the polymer precipitates from the solvent, or the solvent evaporates by the exo-energetic chain growth effect or by reducing the pressure [67]. Conducting the reaction in a solvent reduces the mixture’s viscosity, increasing the monomers’ mobility and making them easier to meet. The solvent polymerization method was used to synthesize the polyurethane network using octaaminophenyl-POSS (Oap-POSS) containing eight aminophenyl groups. MOCA was used as the chain extender. The synthesis was carried out by the prepolymer method, and TDI, PPG, and MOCA were used as isocyanate, diol, and chain extending components, respectively. Oap-POSS was introduced in the DMF solution, which was removed after the reaction under reduced pressure [83].

### 2.3. Blending

Blending is a physical process when POSS is mixed with the polymer without a chemical reaction. POSS is functionalized only with the nonreactive groups that enhance compatibilization between the POSS and a polymer (Figure 1H). This way of POSS incorporation offers several advantages, mainly technological, such as ease of processing and versatility. On the other hand, interactions between POSS particles often result in their aggregation [84,85,86,87,88], and the successful dispersion depends on the surface interactions of POSS with polymers, such as van der Waals and hydrogen bonding [84,86,89,90]. Polyurethane and POSS can be blended in solution [87,91], in the melt [92], or via polymerization in the presence of nonreactive POSS [84,91]. 

## 3. Biomedical Applications of PU/POSS

### 3.1. Membranes

Blood biocompatible membranes can be obtained by electrospinning of POSS-PU copolymer. The homogeneous mixture of open cage trisilanolisobutyl-POSS (TSI-POSS) in PTMG was reacted at 110 °C with MDI and chain extended with BDO. The 12% of POSS-PU solution in DMF/THF (*w/w* = 1:2) was electrospun with 0.8 mL/h extruding speed, at 20 kV, and with 20 cm distance between tip and collector. Only 2% of POSS in the obtained membrane had significantly increased contact angle, inhibited bacteria growth, and decreased platelet and protein adsorption. The results are promising; however, cytotoxicity has not been tested. 

### 3.2. Bioactive Forms of POSS

In many cases, due to low surface free energy, POSS molecules are phase-separated [93]. This property is an advantage when obtaining antimicrobial surfaces and products with spontaneous antimicrobial properties that may be used in places when microbes are particularly undesirable, like hospitals, water treatment stations, or in the packaging industry [94]. There are two main ways to obtain antimicrobial POSS: (i) functionalization with ammonium salts and (ii) immobilization of heavy metals. 

**Figure 2:**
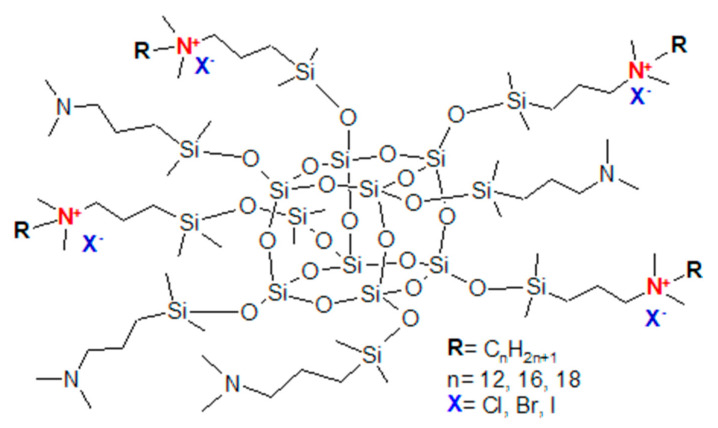
Q-POSS idealized structure. Reprinted with permission from P. Majumdar, E. Lee, N. Gubbins, S. J. Stafslien, J. Daniels, C. J. Thorson, B. J. Chisholm (2009). Polymer, Elsevier.

As antimicrobial agents, quaternary ammonium salts (QAS) are one of the most used cationic disinfectants, mainly because they can kill the versatility of microorganisms like fungi, bacteria, and algae [95]. Moreover, they are harmless to humans and animals [96]. They act as “contact-killers” by breaking through the bacterial cell wall, using a long hydrocarbon chain on an amphiphilic ammonium center resembling a sword blade [97]. First, the hydrocarbon chain is absorbed on the bacterial cell wall due to the electrostatic attraction between positively charged hydrocarbon chain and negatively charged cell membrane. Then it is diffused through the cell wall and absorbed onto the cytoplasmic membrane. Finally, it disrupts the cytoplasmic membrane, and the resulting leakage leads to the cell’s death [98]. The fact that QAS are active for an extended period makes it rational to bond them to another molecule or polymer, obtaining an antimicrobial coating [99]. Despite being useful in microbe-undesirable applications, such polymer gains resistance against biological corrosion as well [100]. With its relatively small molecular size and the possibility of multiple QAS substituents, functionalization of POSS results in increased charge density, thus enhanced antimicrobial properties. Various types of POSS with variable quaternization were examined by Chojnowski et al. and found to be effective against gram-positive and negative bacteria [96]. The methods and ways of synthesizing QAS-functionalised POSS (Q-POSS, Figure 2) and their incorporation into polymers have been summarised by Simionescu et al. [94]. Also, the array of Q-POSS was synthesized by Majumdar et al. [101]. Their research showed that only the use of low-quaternizated POSS had resulted in coatings with antimicrobial properties. The antimicrobial properties of highly quaternizated POSS were suppressed by agglomeration. Application areas as additives to silicone paint commonly used in hospitals [96,102], to methacrylate glass [103], as ceramic water filters [104], and as anti-biofouling coatings with polydimethylsiloxane for the marine industry [102] have been presented, to name a few. Siddiqui and York have examined the toxicity of quaternary silsesquioxane on rats and stated that oral administration as high as 1000 mg/kg/day did not produce teratogenicity or other indications of developmental toxicity [105]. Q-POSS copolymers with polyurethanes seem to be a promising direction, especially as biodegradable materials used in biomedical fields [15,106,107,108,109,110,111]. 

A similar approach was applied by Chawla et al. in which two types of POSS/polyurethane copolymers have been used to support each other in wound healing applications. The first copolymer was biodegradable POSS-PCL (POSS incorporated into poly(caprolactone-urea)urethane) containing poly(hexamethylene carbonate), which was protected by the second copolymer, biostable POSS-PCU (POSS incorporated into poly(carbonate-urea)urethane). The outer layer of POSS-PCL was designed to protect the inner, biodegradable POSS-PCU, and thus it was impregnated with silver as an antibacterial agent. As mentioned earlier, both copolymers are known for their excellent microporous topography and great distribution of pores that allowed cellularization with adipose tissue-derived stem cells (ADSCs) within a few days, making the composition well-suited in wound healing applications. Silver was applied as nanoparticles, and authors declared no cytotoxicity of the composite, although no immobilization of silver was secured. The first scientific reports about silver germicide properties come from the XIXth century, but they are known from ancient times. Silver nanoparticles are of much interest because of their significant antimicrobial activity [111,112,113]. Unfortunately, they are not as safe as the QAS as their cytotoxic and genotoxic effects have been reported [114]. Immobilization of silver particles may help to reduce those undesirable side effects. Fahimeh et al. used hydrochloride salt of octa-aminopropyl POSS as an organic-inorganic crosslinker in PEG-based hydrogel to immobilize silver nanoparticles [115]. The nanoparticles were synthesized in situ in a hydrogel matrix by reducing silver ions by NaBH4 and then entrapped with POSS ammonium salt via hydrogen bonds. The surrounding hydrogel acted as a matrix for nanoparticles resulting in their fine and homogeneous distribution. The resulting nanocomposite was found to be effective against Gram-positive S. aureus and Gram-negative E. coli. PU/PEG-based thermoplastic hydrogel, copolymerized with POSS, was synthesized by Wu et al. in the presence of silver nitrate [116]. POSS and PEG thermodynamic incompatibility determined microphase separation, which resulted in the synthesis of hydrogels in a swollen state that had entrapped silver ions. The compact inner structure of hydrogel caused a slow release of silver ions resulting in the extended 14-days period in which the formation of E. coli biofilm was suppressed.

**Figure 3:**
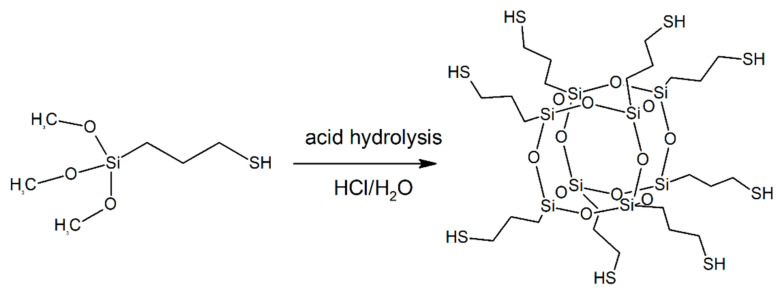
Cu-chelating POSS synthesis and structure.

Not only silver is used as a metallic antimicrobial agent. Copper is also known for centuries and in ancient times was used to treat wounds and sterilize water. Cu ions sterilization properties are due to the generation of hydroxyl radicals that oxidize bacterial proteins, lipids, DNA, and other (macro)molecules [117]. Noteworthy, numerous reports about copper nanoparticles for antimicrobial applications have been published recently [118,119,120,121,122,123,124,125,126]. The POSS particles can also be used as an immobilization agent for copper nanoparticles (Cu-NP). An example is Cu-chelating POSS synthesized from (3-mercaptopropyl)trimethoxysilane (Figure 3), thus bearing eight thiol groups, able to chelate copper and to react with isocyanate yielding thiolurethanes [127]. The tests of antimicrobial properties in the presence of common hospital microbes showed that the materials were equivalent or superior to silver-containing fibers. Authors fabricated polyurethane, epoxy, acrylic, and polyester coatings containing Cu-NP that can be applied on aluminum and stainless steel [128].

**Figure 4:**
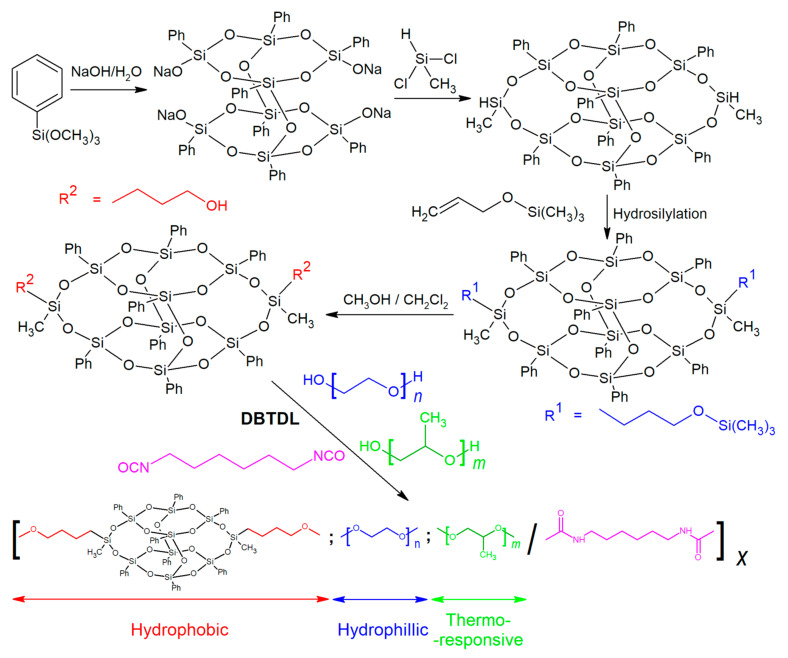
Schematic representation of BPOSS synthesis and the building blocks of obtained polyurethane. Reprinted with permission from Han, Y.; Xu, C.; Shi, H.; Yu, F.; Zhong, Y.; Liu, Z.; Loh, X.J.; Wu, Y.L.; Li, Z.; Li, C. (2021) Chem. Eng. J., Elsevier.

POSS high functionality, self-assembly tendencies, and biocompatibility can meet in drug delivery applications [129]. POSS compounds can help with the poor solubility in water/peptides, which can either weaken or stop the medical effect of the drug. Furthermore, drug loss due to tearing in ocular infections can be a severe problem. POSS-derived micelles or dendrimers that can transport drugs into various media may be a helpful tool in this field. Taking advantage of the POSS hybrid character, micellization is feasible. Silsesquioxanes may also prevent the drug from leaching by impeding the movement or binding with mucine. This was applied in a POSS-containing copolymer that self-assembled into a micelle around Amphotericin B (AMB), a drug poorly soluble in lipids, used to treat fungal keratitis. First, a bi-functional POSS (BPOSS) was obtained by a four-step reaction, starting from phenyl methoxy silane condensation in NaOH solution (Figure 4). In this way, the partially condensed double-decker silsesquioxane (DDSQ) was obtained, bearing four sodium alkanoate groups on both ends. Those groups were then capped by dichloromethylsilane, and a hydrosilylation reaction was conducted with allyloxytrimethylsilane. Finally, the O-Si bond on two capping groups was hydrolyzed to obtain two hydroxypropyl groups attached to POSS inorganic core opposite each other. Such amphiphilic POSS may be used to control the motion of the macrochains [130] in e.g., PEG/PPG block copolymer. Then it was used in PEG/PPG urethane block copolymer as a comonomer. The reaction of copolymerization was urethanization between hexamethylene diisocyanate (HDI) and three various polyols: poly(ethylene glycol) (PEG), poly(propylene glycol) (PPG) or BPOSS in three concentrations–0.5, 1, and 2 wt%, and molar PEG:PPG ratio ~2:1. The polymer was dissolved in water with various concentrations of sodium hyaluronate and 1,6-diphenyl-1,3,5-hexa-triene (DPH), and micellization with AMB was done in DMSO drained by the dialysis. The micelles were ~115 nm in size, and drug loading efficiency was ~29%, not dependent on BPOSS concentration. The authors found no cytotoxicity of the produced drugs, POSS micelles did not increase the intraocular pressure (IOP), and comprehensive studies on mice infected with fungal keratitis showed that treatment had better efficiency than currently available drugs [131]. 

### 3.3. Cancer Treatment

**Figure 5:**
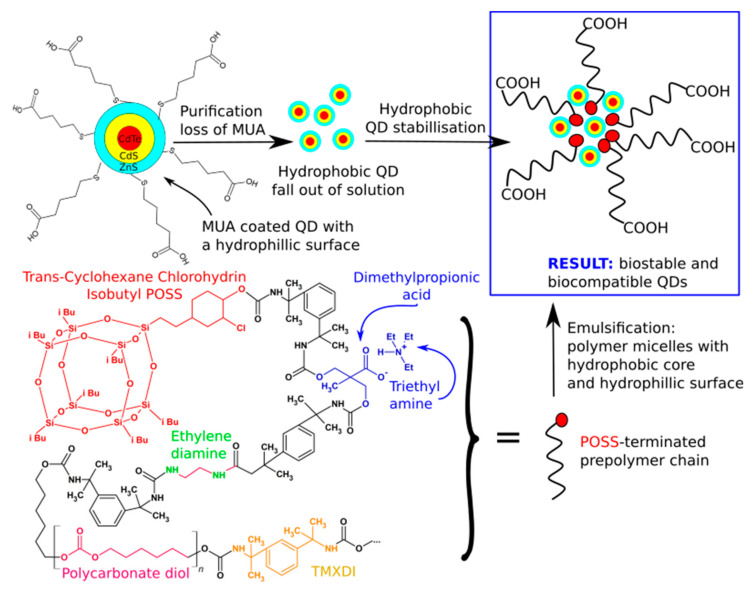
Schematic representation of the POSS terminated prepolymer and emulsification of QDs. Rizvi, S.B.; Yang, S.Y.; Green, M.; Keshtgar, M.; Seifalian, A.M. (2015) Bioconjug. Chem, ACS.

By the year 2015, cancer had caused 15.7% of deaths [132]. POSS composites can be used in both methods of combating cancer-therapy and diagnostics. In the first case, materials containing POSS and carbon nanotubes have been developed. The nanotubes were covered with POSS-PCL composite, thanks to which a biocompatible surface was obtained. Such materials show strong near-infrared absorption, and while illuminated, emit a large amount of thermal energy. This effect is synergistic due to both nanotubes and POSS; therefore, these materials can be used to destroy cancer cells [25]. POSS has also found its application in the field of in vivo imaging, a general group of cancer diagnostic techniques. Here POSS-PCU is also used as a biocompatibillizer of quantum dots (QDs) [133]—fluorescent nanoparticles that potentially could be used in various bio-imaging applications replacing organic dyes and fluorescent proteins. However, their use is limited because of the toxicity of their cadmium salts-based cores. In 2015 Seifalian et al. reported on encapsulation of CdTe/CdS/ZnS QDs using POSS-PCU oligomers, stabilized in the form of 33 nm micelles, as evidenced by the DLS technique (Figure 5). The QDs were functionalized by mercaptoundecanoic acid (MUA), and the POSS-PCu copolymer was composed of tetramethylxylylene diisocyanate (TMXDI, polycarbonate diol, trans-cyclohexane chlorohydrin isobutyl POSS, and dimethyl propionic acid (DMPA) to form an isocyanate-terminated prepolymer [134].

### 3.4. Biomaterials

In biomaterials, POSS plays an essential role as a bio-compatibilizer due to bearing multiple Si-O bonds. Molecules containing this bond, especially when combined with hydrocarbons, are usually biocompatible because of Si-O bond-induced chemical stability and surface properties (low surface energy and hydrophobicity) [7]. In addition, because the polydimethylsiloxane polymers have weak mechanical properties, POSS is often used as a reinforcing agent. Notably, POSS tend to migrate into the surface, thus giving desirably cytocompatible and non-toxic properties to the place where they are very much needed [93,135,136].

#### 3.4.1. POSS-PCU

Polyurethane is widely used in the field of biomaterials, especially for blood-compatible materials. The applications range from catheters to artificial hearts, and polyurethane is the third most commonly used polymer for vascular surgery [137,138,139]. However, their thrombus formation resistance is limited, and this leads to dangerous obstruction of the grafts. Among polyether and polyester-based polyurethanes, the most biostable are poly(carbonate-urea)s, in which hard segments were composed of urea, and soft segments were polycarbonate [140]. However, the thrombogenic resistance of PU-based materials needs to be improved. 

Here is where POSS can come into the light. Because of its variable surface tension, the use of POSS in those materials resulted in platelet and fibrin repulsion and significantly increased thromboresistance without elasticity mismatch and intimal hyperplasia effects associated with materials containing silicon [7,141]. The studies concerning the anti-thrombogenic effect of POSS-PCU hybrids revealed that three phases had formed on the surfaces of the compound. The third phase was crystalline with domains 5–20 µm, while the two phases had formed a “pebble stone” structure whose domains were 200–500 nm in size and 100–200 nm in height. Either the existence of this structure or the presence of the POSS building blocks in hard segments, diluting the number of hard segments in the polyurethane matrix, caused the effect of high wetting angle hysteresis, which seems to be a good measurement of protein/platelet repulsion [141]. The hysteresis was to some extent independent of POSS concentration, suggesting that mainly the surface topography caused by POSS reorientation played a key role. 

**Figure 6:**
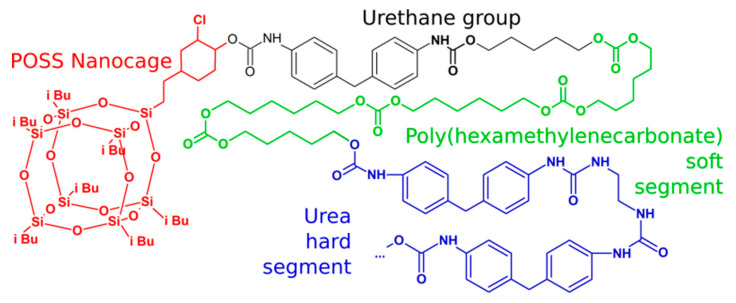
Structure of original PCU-POSS. Reprinted with permission from Rahmani, B.; Tzamtzis, S.; Ghanbari, H.; Burriesci, G.; Seifalian, A.M. (2012) J. Biomech. Elsevier.

POSS-PCU was synthesized from MDI, trans-cyclohexanechloroydrinisobutyl POSS, and poly(carbonateurea) glycol, and a mixture of 40:1 (by wt.) ethylenediamine: diethylamine as a chain extender (Figure 6). In the beginning, POSS was dissolved in polyol at 125 °C and cooled to 60 °C. Then MDI was added, and the reaction ran at ~80 °C for 90 min to obtain a prepolymer. Then DMAC was added to dissolve it, and the amine mixture was introduced at 40 °C [142]. The high biocompatibility of this material allowed it to be successfully taken to the clinical tests as a replacement for trachea, tear duct, and vascular bypass grafts [106,143]. 

Most of the further investigations on POSS-PCU were about modification techniques. Auspicious results have been reported recently concerning argon plasma surface modification of POSS-PCU [144,145]. The scaffolds from non-treated POSS-PCU, Ar plasma-treated POSS-PCU, and commercial Medpor^®^ porous polyethylene have been investigated by various methods. Protein adsorption, Human Dermal Fibroblast seeding, and assessment of their morphology, adhesion, growth, and secretion of collagen and VEGF have been performed. Furthermore, seeding of macrophage cell cultures, assessing their adhesion and growth, and immune response in vitro and in vivo analysis on laboratory mice were conducted. Protein adsorption, vital for cell adhesion onto the surface [145], was comparable between non-treated PU and Medpor but a few times higher in plasma-treated PU. Plasma-modified PU was less stiff, rough, and hydrophobic than Medpor, which determined higher protein conformation (tested for fibronectin and vitronectin, but not for albumin, fibrinogen, and plasma proteins), contradicting literature statements that hydrophobic and rough surfaces are more promising [146,147]. Higher protein absorption influenced all other assessments in which Ar plasma-treated PU was exquisite, and its higher tissue integration potential was confirmed in-vivo after 12 weeks of subcutaneous implantation. Despite observed differences in the in vitro macrophage responses between all three scaffolds, no differences were observed in the in vivo recruitment of macrophages to alter the immune response. Ar plasma treatment of POSS-PCU revealed this technique’s potential to improve tissue integration [100,101]. In the case of biodegradable POSS-PCU-PCL biomaterials [148], argon plasma can be used to sterilize those materials, which cannot be sterilized by standard FDA approved autoclaving technique [149,150]. 

**Figure 7:**
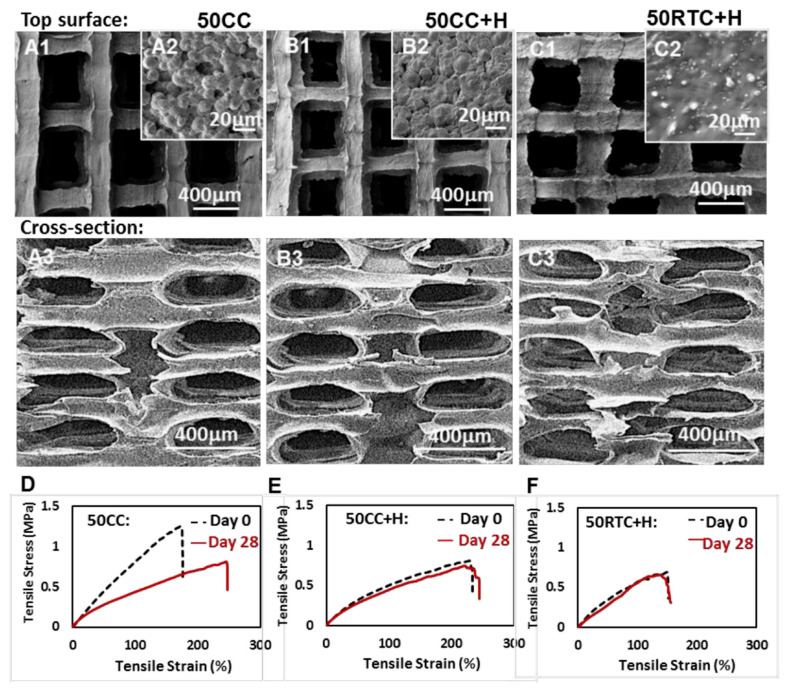
Stiffness memory as visible at stress-strain curves at day 0 and 28 of incubation (**D**–**F**) and corresponding structures of PUU-POSS scaffolds (**A**–**C**) obtained at different conditions (samples names assigned above the figure). Reprinted with permission from Wu L.; Magaz A.; Maughan E.; Oliver N.; Darbyshire A.; Loizidou M.; Emberton M.; Birchall M.; Song W. (2019); Acta Biomaterialia, Elsevier [156].

When the material is not being rejected, one can focus on the mechanical properties of the implant. The synthetic scaffolds and implants are often designed as stronger and stiffer than the surrounding tissues due to the focus on optimizing mechanical stability without paying attention to adapting the implant in an organism-environment. Due to the apparent difficulties with the diversity of living organisms within even one species, most tests are performed in vitro, not in vivo. Nevertheless, a mismatch of the mechanical scaffold properties in living tissue can result in remodeling of the host tissue, bones becoming less dense and weaker, cartilages remodeling its stiffness or formation of fibrous scar [151,152,153]. A slight mechanical mismatch was observed in tracheal implants of POSS-PCU that were rejected in respiratory system rabbit models [154]. In addition, the mechanical scaffold properties can influence fibroblast infiltrating the wound and orientation of the extracellular matrix [155]. POSS-PCU telomerised on both ends with polyhedral oligomeric silsesquioxane was applied to obtain thermoresponsive non-biodegradable scaffolds by the 3D-TIPS technique. It is a unique technique combining 3D print and thermal-induced phase separation [156]. This approach makes it possible to easily adjust the shape of the stent to the medical case, simultaneously reducing major drawbacks of TIPS, namely the non-uniform porous structure and 3-D technique drawbacks, such as low resolution and limited availability of the filament types. The uniform porous structure was obtained by sacrificial printing the microchannels enabling proper heat exchange. Moreover, the thermodynamic control of the phase separation creates a possibility to adjust stiffness and porosity by setting different processing temperatures and post thermal treatment conditions, reducing the mechanical tissue-implant mismatch. The obtained scaffolds showed a “stiffness memory” (Figure 7), a softening effect as the temperature of melting the soft segments was close to body temperature (Tm = 45 °C). Two stages drove the softening: thermodynamic phase transition due to melting the semicrystalline soft domain, and local chain self-assembly of the nanostructures. This effect and hierarchical porous structure modulate tissue ingrowth and reduce in-vivo inflammation in a rat model for up to 12 weeks. Furthermore, the polarization towards the macrophage M2 phenotype was enhanced; thus, a regenerative model of scaffold tissue interaction was obtained [156].

#### 3.4.2. Biodegradable POSS-PCL

Biodegradable PU-POSS materials are usually composed of biodegradable polyol, mostly polycaprolactone diol (PCL). An example can be a polymer synthesized using HDI (hexamethylene diisocyanate) and PHI-POSS (1,2-propanediolisobutyl POSS). Additionally, PCL was used as a biodegradable diol, synthesized from either glycolide ((PGCL)_1k_) or D,L-lactide ((PLCL)_1k_), copolymerized with ε-caprolactone and butanediol [157]. The reaction was done in the presence of (tin(II) 2-ethylhexanoate) at 140 °C under a nitrogen atmosphere for 10 h. The obtained polyols with various ratios of caprolactone and comonomer (glycolide or lactide) were then reacted with HDI and POSS. Changes in comonomer, caprolactone, and POSS ratio resulted in the tailorable elasticity of obtained thermoplastic polyurethanes. The crystallinity of POSS was relatively high, which was evident on WAXD diffractograms, and it has been observed in HDI/PHI-POSS compositions due to the high rate of their mutual reaction [34].

#### 3.4.3. Circulatory System Implants

The requirements for tissue engineering for the vascular grafts have been described in detail by Boffito et al. [158]. The implant must replicate the properties of the ECM (extracellular cardiac matrix) in terms of geometry, mechanical properties, and interaction with cells. Therefore, the desired material should be biocompatible, biomimetic, and biodegradable.

In detail, the responsibility of the implant patency is mainly on the side of a suitable charge, energy, wettability, and topography [137,159,160]. Furthermore, the material mechanical properties must be easily tailorable, depending on the application (in case of the cardiac tissue: modulus 10–50 kPa, tensile strength 3–15 kPa, and strain 22–90%), with high reproducibility, especially it should have sufficient modulus without being too stiff [158,161,162,163]. The porosity and promotion of cell growth and attachment should also be sufficient to allow tissue infiltration [164,165]. Moreover, the material should degrade into non-toxic products at the space-time when there is no further need for mechanical support. Because of it, slowly degrading materials perform better than fast degrading [166]. Finally, it should be characterized by non-activating chemistries; they have to reduce platelet and white blood cell activation [137,165,167]. 

In another way, the foreign body response reaction begins with protein adsorption due to exposure to the body fluids. The protein layer is formed on the implant surface, which attracts the cells of the immune system. The macrophages recognize it as a foreign invader and make attempts to devour it. Because the implant size is much bigger than the cells themselves, they fuse to form foreign-body giant cells (FBGCs) and release signals to attract the fibroblasts [168,169]. To overcome this problem, the implant surface shall be treated to prevent forming the non-specific protein coating by producing a non-fouling surface which makes the material invisible to the immune system. To provoke a proper biological-material interaction, the surrounding tissue incident cells must be provided with attached integrin ligands and cytokines [168]. This is a biomimicry approach, as the material communicates with the tissue with understandable biological language. Recently, the strategy to fabricate vascular tissue implants has shifted towards biomimicry and in vivo endothelialization [137].

##### Stents

Four main types of endovascular stents can be distinguished [170]:

- Bare metal stents (BMS),
- Drug-eluting stents (DES),
- Coated stents,
- Graft-covered stents.

Despite significant advances in the stents field, the main problem with their development is still thrombosis/restenosis response [171], which affects 25 to 50% of all vascular interventions. In those cases, repeated angioplasty and the use of long-term anti-thrombotic medication are the only solutions [170]. Distinguishing against the time elapsed after the intervention, there are three models of thrombosis: early-stage (EST < 24 h), sub-acute (SAT < 30 days), late state (LST < 6 to 12 months), and very late state thrombosis (VLST < 1 year). The primary mechanism starts with the wall injury and exposition of the endoluminal layer, which releases tissue, collagen, and von Willebrand factors. It promotes platelets’ adhesion, which activates the release of serotonin, adenosine diphosphate, and thromboxane A2 that activate more platelets. Then conversion of prothrombin to thrombin causes transformation of fibrinogen to fibrin and stabilization of thrombus. The further release of cytokines, pro-inflammatory mediators, and growth factors result in the proliferation of smooth muscle cells (SMC) to the intimal layer and extracellular matrix production, and so-called intimal hyperplasia (IH) [170]. Drugs disrupting this process-anti-thrombotic drugs-have to be delivered for an extended time with carefully dosing. It gave birth to the second generation of stents eluting those drugs during the degradation of the drug-containing polymer coating. This approach provides a sustained, controlled, and predictable rate of drug elution. Nowadays, one can calculate the drug elution rate in diffusion, swelling, and degradation releasing systems [21]. Unfortunately, studies have shown that the DES is prone to late stent thrombosis, most probably due to allergic reactions to the bio-degradable polymers localized at the vessel wall [172]. Clinical studies revealed that thrombosis risk is highest when DES are used for complex lesions [173]. The more recent approach in which POSS plays a crucial role are the covered stents. They usually have a thin membrane sleeve that either covers the stent surface against the vessel wall or completely covers the stent in a sandwich-like configuration. Farhatnia et al. had outlined the criteria that covered stents should fulfil [170]:

- May be folded or compressed for efficient delivery,
- Have high flexibility for easy maneuvers during delivery and deployment,
- Possess predefined expandability rates and strength with negligible recoil after implantation,
- Low deployment pressure,
- High burst resistance (>500 mm Hg),
- Low water permeability (1 mL/cm^2^ min^−1^ at 120 mm Hg),
- Controllable with the use of magnetic resonance imaging (MRI),
- Haemocompatibile, not causing allergenic reactions,
- Modified biomimetic surface with biomimetic peptides, antibodies and growth factors, nanomaterials.

The effortlessly tailored properties of polyurethanes and biocompatibility of PTFE have decided their general use in this field. However, their main drawback is low oxidation resistance. To overcome these problems, POSS copolymer with poly(carbonate-urea) was synthesized, which had fruited with a family of non-biodegradable and bioabsorbable polymers that meet the essential criteria in the development of covered stents. This composite is biocompatible, provides excellent endothelialization support, is non-toxic, causes no inflammatory response, being simultaneously strong and durable [170]. Furthermore, according to GPC, SEM, and stress-strain studies, POSS has a shielding effect on the soft segments of the polyurethane and protects against hydrolysis and plasma protein fractions and other forms of degradation, not much affecting mechanical properties like elasticity [171].

Moreover, POSS can be further modified by incorporating other functional groups that modify the copolymer chain or surface. The functionalization may also be done from the metallic stent site for a better junction between polymer and metal. It is beneficial to apply polymer by spray technique, which ensures better folding and flexibility of the stent. Electrospray or ultrasonic sprayed film must cling well because the exposed bare metallic stent surface has thrombogenic potential, mainly by releasing Ni ions [171].

**Figure 8:**
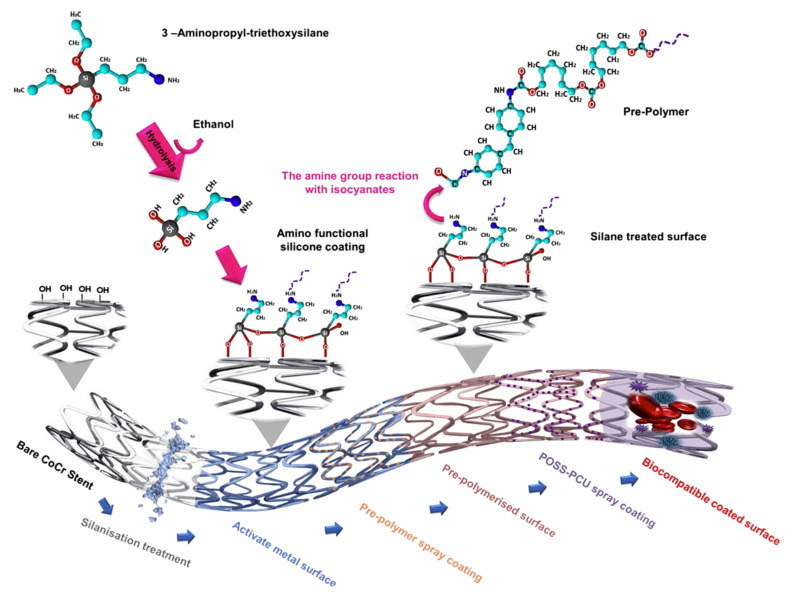
BMS stent pre-treatment and POSS-PCU spray coating procedure to obtain a biocompatible coated surface. Reprinted with permission from Farhatnia, Y.; Pang, J.H.; Darbyshire, A.; Dee, R.; Tan, A.; Seifalian, A.M. (2016) Nanomedicine: Nanotechnology, Biology, and Medicine, Elsevier [174].

The surface modification technique may be based on anodization of NiTi alloy in sodium nitrate MetOH solution and 1-h heat treatment at 600 °C. The resulting titanium oxide coating is then subjected to sodium hydroxide to saturate the surface of the oxide with OH groups entirely and then silanized by 3-aminopropyltriethoxysilane in acetic acid for 15–60 min at room temperature. Then, cross-linking may be done at the elevated temperature (110 °C, 20 min). As a result, the metal surface is covered with Ti-O-Si bonds with the aminopropyl end that can react with PCU/POSS after primed with the prepolymer and covered with PCU/POSS hybrids using ultrasonic atomizing spraying technique. The chemistry of the process is shown in Figure 8 [171].

Silanisation can also be done for other types of BMS, for example, the CoCr type. An important parameter is the thicknesses of the polymer covering membranes. It was found that the optimal thickness in the case of POSS/PCU is 20 µm; above this value, the stent recoil percentage is drastically increasing [174].

The second approach to functionalization are biomimetic surfaces that are getting more and more attention in the new compositions for stent coating. A remarkable advance was the composites’ ability to capture anti-CD34, the epithelial progenitor cells (EPC) antibodies, crucial for endothelialization [175]. Authors used at first a blend of amino-functionalized silica with POSS-PCU copolymer [176]. To successfully capture the antibodies, a linker molecule from N-(3-Dimethylaminopropyl)-N’-ethylcarbodiimide hydrochloride (EDC), N-hydroxysuccinimide (NHS), and succinic acid has been synthesized in phosphate-buffered solution (PBS) using a roller mixer [177]. The EDC-NHS linker was able to bond to amino groups in fumed silica and peptide motifs in antibodies. It was applied in PBS solution on POSS-PCU disks and left in darkness for 3 h. Then mouse anti-CD34 concentrate in PBS was applied; the disks were stored in darkness and temperature 4 °C, for 24 h [178]. The antibodies displayed increased cell adhesion on POSS-PCU, and although they were non-specific to EPCs, the hemocompatibility increased. 

PU-POSS composites with their ability to separate into POSS microdomains were utilized in another type of stents, namely polymeric shape-memory stents. Their ability to self-expand in the pre-set temperatures could be applied in stents expanding in the body temperature range. Those materials contain a two-phase shape transition. In the first phase, they may be fixed in a temporary shape, while in the second phase, the polymer is stimulated (usually by heat) to recover its permanent shape. The thermally-induced shape memory polymers are obtained by forming cross-linked networks while maintaining the ease of chain conformation between adjacent cross-linking sites [179]. Thus, self-expanding could omit the BMS core from the stents resolving most problems they are subjected to.

**Figure 9:**
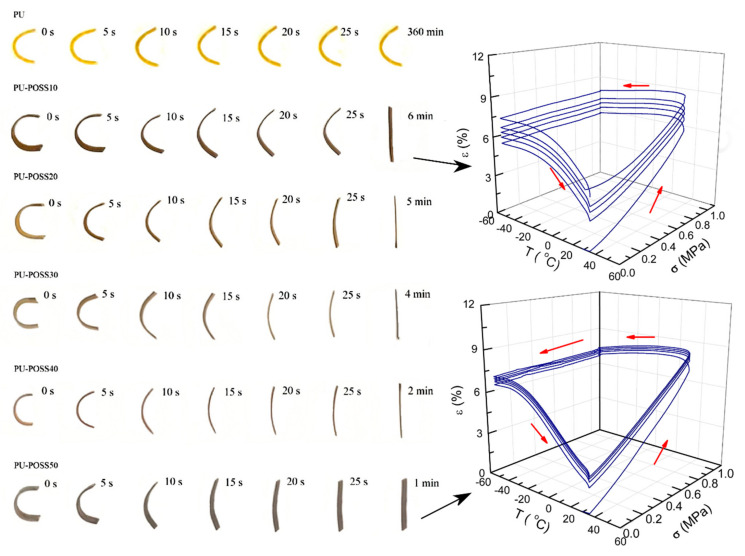
(**Left**): Shape recovery of samples with loadings of POSS from 0% to 50% (**Right**): changes in strains of one–way shape memory cycles for the lowest and the highest loadings of POSS. Reprinted with permission from Zhao, B.; Xu, S.; Adeel, M.; Zheng, S. (2019) Polymer, Elsevier.

On the other hand, most polymers gradually lose mechanical properties after application due to fatigue failure [180,181]. Efforts are being made to resolve this problem by employing nanotechnology. The synthesis of diolPOSS (bearing seven phenyl nonreactive groups and one reactive with two identical primary hydroxyl groups on ester chain) and its incorporation into MDI-BDO-PTMG polyurethane matrix (4,4′-diphenylmethane diisocyanate, butanediol, polytetramethylene glycol) resulted in a linear polyurethane in which POSS and BDO played a role of a chain extender. The POSS-containing segments had self-arranged into spherical 10–20 nm domains that promoted physical cross-linking, and thus the copolymer displayed good shape memory properties contrary to unmodified PU (Figure 9). Furthermore, the mechanical properties and glass transition temperature have increased [179]. The most profound effect was obtained for a rather generous 50 wt% quantities of POSS, but in the case of eight functional octakis(dimethylsilyloxy)-POSS, 3.8 wt% was enough to obtain shape memory with high shape recovery properties [182]. However, the obtained polyurethanes were crosslinked chemically and thus not thermoplastic. In the case of different morphologies, the POSS can act inversely with the increased loading. It is so in the case of polyurethane PPG-MDI-BDO (PPG-polypropylene glycol) modified with DDSQ-diol (double-decker silsesquioxane). The authors obtained polyurethane-DDSQ hybrids in which POSS was used as a chain extender in wt% ranging from 8.0 to 45.3. The fastest recovery was visible for the lowest loadings of POSS. 

##### Valves

Since the 1950s, polyurethane valves have been in use. However, they lack long-term durability [183]. The main share in bioprosthesis failures holds calcification, by predisposing the leaflet to the structural damage and rupture of the cusps [184,185]. POSS-PCU with telomerising trans-cyclohexanechlorohydrinisobutyl-POSS was used as a material for the production of the valve, and the mechanical and surface resistance was measured before and after the exposition to calcium solution in a heart imitating pulsatile pressure system for 31 days and 4 × 10^7^ pulses. X-ray, microscopic, and chemical investigations were performed to follow the signs of calcium deposition. The chemical analysis evidenced that calcification was significantly reduced in POSS-PCU contrary to pure polyurethane and bovine pericardium [184]. The key to inhibiting calcium deposition appears to be a high wetting angle. Octavinyl polyhedral silsesquioxane (OVS) was used as a surface modifier during the grafting of heparinized carbon nanotubes (HCNT) on PU surface [186,187]. Grafting of vinyl-POSS on PU/HCNT was done after ionized O_2_ plasma treatment, which formed hydroperoxide groups on the surface, and OVS was able to chemically bond with them. Bonding was visible as a reduction of 1380 cm^−1^ band attributed via FTIR-ATR analysis to -OOH groups. The presence of HNCT had a synergistic effect on OVS grafting since EDS maps showed its higher concentration on PU/HNCT than on pristine PU. Plasma-induced POSS grafting had expanded the average surface roughness substantially and wetting angle up to 165°, which resulted in lower calcification and lower platelet adhesion; however, in the last case, plasma treating alone seemed to be essential. 

#### 3.4.4. Scaffolds

##### Nervous System

Another application of POSS-PCU are synthetic nerve conduits. They were obtained by subsidizing PCU with the RGD peptide (arginine-glycine-aspartamic acid) [110] and are currently in the clinical trial phase to confirm their ability to regenerate nerves at a distance greater than 30 mm, which was unachievable before. POSS-PCU-PCL doped with multi-wall carbon nanotubes were found to be homogeneous and electrically conductive. To achieve a high degree of homogenization, sonication, and functionalization with sodium dodecylbenzenesulfonate (SDBS) were applied. Potential applications focus on brain damage regeneration, prosthetic devices, and brain implants [187]. Instead of carbon nanotubes, one can also use graphene to obtain an electrically conductive polymer. The graphene was dispersed in DMAC by sonication for three hours, and then POSS-PCL was added to obtain a composite solution. Next, hydrazine hydrate was added to reduce any GO (graphene oxide) emerging groups. After five hours of mixing at 100 °C, the composites were poured into aluminum substrates and soft-baked for 12 h. SEM and AFM analysis showed good homogeneity of graphene. Ultra-low percolation threshold at 0.8 wt% of graphene was observed, and in the case of 4 wt% the conductivity was nine orders of magnitude better than for pristine POSS-PCL. Schwann cells were seeded on the materials and AlamarBlue test was performed indicating better cell viability and proliferation in materials containing graphene [108].

##### Skeletal System

Two types of POSS: octa(3-hydroxy-3-methylbutyldimethylsiloxy) POSS (OCTA-POSS) and 1,2-propane-dioliso-butyl POSS (PHI-POSS), were used to modify flexible polyurethane foams as potentially active bone scaffolds. The foams were produced with aliphatic hexamethylene diisocyanate (HDI), which is less toxic than MDI, and a mixture of two polyetherols: sorbitol-based and glycerine based one, with POSS loadings varying from 0 to 15 wt% and water as a blowing agent. The compressive strength of the foams at 40% deformation was highest for small amounts of POSS, deteriorating with further POSS loadings and resulting in POSS crystallization. Crystallization was visible via WAXD in the case of PHI-POSS, and the compression strength of those samples was 150% higher than reference in lowest loading and 75% lower in highest loading. Despite no evidence of crystallization, OCTA-POSS also followed this trend, reaching ~200% and 150% increase for lowest and highest loadings. After four-week incubation in simulated body fluid (SBF), scanning electron microscopy showed numerous spherical hydroxyapatite (HAp) granules that were directly size- and amount-dependent on POSS concentration in PU matrix. Energy-dispersive X-ray spectroscopy revealed a Ca/P ratio varying from 1.54 to 1.63, indicating the presence of well-defined HAp. After preparation, the adherent established cell line of human osteoblast U-2 was placed on materials and cultured for 24, 48, and 72 h in a laminar flow chamber. The cell cycle and apoptosis with Annexin V were evaluated. Materials negatively affected the cell viability, but with higher loadings of POSS, the cytotoxicity was reduced, as a noticeably smaller percentage of dead cells was observed in the case of modified foams compared to reference [13]. 

##### Liver

POSS-PCU-PCL was also used as a porous scaffold for liver restoration [188]. The pores were induced by glucose, sodium bicarbonate, and sodium chloride particles (glucose appeared to be the best solution) and seeded with hepatocytes (HepG2). The SEM microscopy showed suitable cell attachment, and a steady albumin secretion was registered, while in the case of control nonporous material, there was a steep decrease after seven days. Unfortunately, those interesting findings remained at this initial stage of research. 

##### Respiratory System

One of the most challenging procedures is replacing the upper airway epithelium with exogenous cells [189]. POSS-PCU trachea scaffolds were compared with collagen I-based scaffolds, as well as with decellularised dermis and trachea. The bioengineered decellularised dermis was the most promising due to collagen IV and laminin retention, which appeared to be a key mediator of airway epithelial cell attachment and proliferation, and integrins α2 and β1 are also important mediators of this process. It is consistent with the role of integrin α2/β1 as a receptor for both proteins [190]. In-vivo assessments for laryngeal cartilage scaffold were done with pigs. The scaffolds were fabricated using two layers of POSS-PCU, solid and microporous. In both cases, POSS-PCU solution in DMAC was prepared by prepolymerising MDI, trans-cyclohexanechloroydrinisobutyl POSS, and poly(carbonateurea) glycol, and chain extension was performed with DMAC solution of ethylenediamine. The obtained 18% POSS-PCU solution in DMAC was poured into the mold, and one part was dried at 65 °C for 18 h to remove DMAC. The new portion of the solution was mixed with 50 wt% NaHCO_3_ particles (100–150 µm in diameter) and NaHCO_3_ with DMAC were extracted with water. The obtained product was seeded with human bone marrow-derived mesenchymal stem cells (BM-MSC) at 200,000 cells/m^2^ in Bronchial Epithelial Growth Medium (BEGM) incubated for 48 h. Then the medium was changed into Minimal Essential Medium (αMEM), and primary human airway epithelial cells were seeded with density 1.5 × 10^6^/m^2^ and, after 72 h, orthotopically implanted in place of a previously prepared laryngeal defect. One from eight pigs had expectorated its implant and was terminated early–after two weeks. Endoscopy was performed at weeks 1, 2, 4, and 8 showing a smooth appearance suggesting mucosal regeneration, and mucosal brushing showed cells with epithelial cell morphology and no phenotypically-human cells (beyond four weeks). Full extrusion of implants was complete at the time of termination (8 weeks) [151]. The successful use of exogenous (for pigs) human bone marrow and epithelial cells is promising. However, no longer than eight weeks of tests have been done.

##### Cartilages

A solvent-melt method can be used to produce artificial meniscus material. It was synthesized by using monofunctional POSS that was telomerising polyurethane chain. It was found that polyurethane chains capped with POSS have better mechanical properties, oxidation, and calcification resistance. Furthermore, the cell proliferation analysis done by fluorescence tracking showed that many cell cultures were proliferating in-vitro, and fusing proceeded more quickly than in the control sample. The POSS-capped polyurethane scaffold showed well in vivo biocompatibility; however, fixing the composite in the meniscus prosthesis is still challenging [191].

## 4. Characterization

### 4.1. Mechanical Properties

#### Adhesion Strength

Adhesion strength is an essential factor of covered stents. The broadly used nitinol stents were entirely covered to prevent Ni ions from leaching, facilitating thrombus formation. The proposed technique is based on classical tensometer measurement in which one handle is holding a stripe, manually peeled off from the polymeric coating, and another clamping the stent with an initial distance of 10 mm between the grips. In earlier studies, a tensometer equipped with a 500 N load cell was used to measure the adhesion, and the procedure was based on the ASTM D413 standard, which gives recommendations on how to determine the force per unit width required to separate a rubber layer from a flexible substrate such as fabric, fiber, wire, or sheet metal [192]. The test was conducted with a 5 mm/min crosshead displacement rate to a final extension of 40 mm. The adhesion strength is equal to the mean force recorded during the test [193].

### 4.2. Surface Properties

#### 4.2.1. Wetting Angle/Surface Free Energy

The wetting angle is an essential property of biomaterials. In the case of biostable materials, it should be high to enhance its stability, while in the case of biodegradable materials, it should be low to encourage cells proliferation. Several methods measure contact angle and subsequent surface free energy, such as static and dynamic sessile drop methods, captive bubble angles, pendant drop methods, various Wilhelmy methods, and finally, capillary rise method. Sessile drop is widespread as it is simple and does not need advanced equipment. Surface-free energy and its dispersive and polar components may be measured by comparing *θ* angles for two different liquids like water (highly polar liquid) and diiodomethane (highly dispersive liquid). A syringe pump is used to dispense the sessile drop through a flat tip needle. The sessile drop must be small to reduce the gravitation effect, and typically it has a volume of a few μL. The wetting angle is measured via digital analysis software [106,174]. The angle values >90° *θ* are considered hydrophobic, and values <90° *θ* are considered hydrophilic. 

#### 4.2.2. Scaffold Porosity

The porosity is thought to be an essential factor affecting cell migration into the biomaterial [1]. Therefore, it is analyzed as a comparison of material density and apparent density (2), while apparent density is a simple division of material mass by its external dimensions (1).

(1) $$Apparent\;density\;\left[\frac{g}{cm^{3}}\right]=\frac{Scaffold\;\;mass\;\left[g\right]}{Scaffold\;\;volume\;\left[cm^{3}\right]}$$

(2) $$Porosity\;\left[\%\right]=\left(1-\frac{Scaffold\;apparent\;density\left[\;\frac{g}{cm^{3}}\right]}{Polymer\;bulk\;density\;\left[\;\frac{g}{cm^{3}}\right]}\right)\times 100\%$$

Microscopies like SEM or AFM usually measure surface porosity. In the SEM technique, graphical analysis software shall measure the surface pore size distributions to obtain numeric data. For AFM, the roughness may be quantified by the deviation of the real measured surface from the area measured (an ideally flat surface). 

### 4.3. Biological

#### Macrophage Polarization

Macrophage polarization from macrophage phenotype M1 to a macrophage phenotype M2 shows how a living tissue reacts to the presence of the implanted biomaterial. M1 macrophages produce proinflammatory cytokines, participate in phagocytosis, and, in the case of biomaterials, produce FBGCs. M2 macrophages facilitate proliferation and induce collagen production, associated with wound healing and acceptance of the biomaterial. The assessments of those macrophage types can be done by immunofluorescent staining to detect the presence of the macrophage capillary markers. CD86 and CD68 are the markers of macrophage M1 and CD163 for the M2; also, T-cells may be measured by CD3/CD4. A confocal microscope can be used to capture images and stained cells counted by image analysis software [156]. 

## 5. Processing Methods

### 5.1. Surface Functionalization

Plasma is defined as a gas in an ionized state [194]. Among various plasma types, radiofrequency plasma is widely used in biomaterial surface treatment. It is obtained by passing an electric current through gas at low or high pressure. Plasma surface modification (PSM) is a technique that helps to create a hydrophilic surface of the material that is easier to adhere to for the cells. During the process, the surface topography changes, and some chemical groups are immobilized on the surface, and it has a beneficial effect on cell adhesion and proliferation [16,25,26,27,28]. In the studies described earlier, Argon modification of PU scaffolds was performed by exposing the scaffolds to 5 min using a radiofrequency plasma generator operating at 40 kHz with a gas flow of 0.4 mbar at 100 W. 

### 5.2. Electrohydrodynamic Spraying

The method is helpful in the fabrication of covered stents [193]. The methodology is similar to electrospinning, and the equipment consists of a stainless-steel needle connected to the positive terminal of the high voltage power supply and grounded plate electrode. The polymer solution is released to the needle by an infusion pump. When the electric field is applied, the solution overcomes its surface tension, and the jet is ejected from the needle. Due to the charge deposited on each droplet and solvent evaporation, the jet breaks up into smaller charged droplets collected on the stainless steel substrate [195].

### 5.3. TIPS/3D TIPS

**Figure 10:**
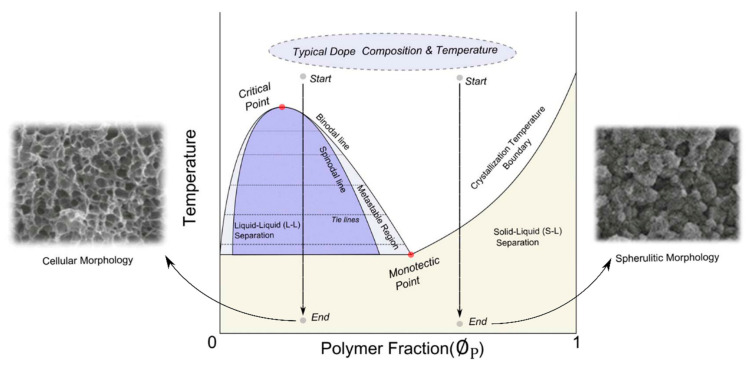
Phase diagram of TIPS process, and comparison of different material morphology obtained. Reprinted with permission from Kim, J.F.; Kim, J.H.; Lee, Y.M.; Drioli, E. (2016) AIChE J., Wiley.

An advantageous technique in scaffold production is TIPS (Thermally Induced Phase Separation) [196]. This technique is based on differences between polymer and solvents’ miscibility. First, one solvent is used to dissolve the polymer, then another, which is not mixing with the polymer, but mixing with the solvent, is added slowly, so the polymer-rich and polymer-poor phases are formed. Then the mixture is cooled down below the second solvent crystallization point and removed by sublimation under vacuum. In this way, the pores are obtained as the polymer-rich phase is left, and in poor polymer phases, voids emerge. Pore sizes and shapes are controlled by the temperature regime and polymer fraction (Figure 10). TIPS technique is vastly used in scaffolds’ production [197]. 

Recent advances in this technique include [198]:

- 3D/TIPS

It is a combination of 3D print with TIPS that can produce a multilevel, hierarchical structure of extracellular matrix (ECM). 3D printing technology is used to obtain a preform from a polymer that is easy to extract (like PEG), which is immersed in a solution of another polymer that is then thermally separated. The solvent is extracted by another solvent, as well as the first polymer preform. The obtained macro/micro scaffold is much-resembling nature [156].

- TIPS and electrospinning

Electrospinning uses an electric current to draw charged threads from the polymer-solvent system or polymer melt. By using electrospinning, nanofibers can be produced, which may be used as a preform in the TIPS technique. By combining these techniques, a network of interconnected channels can be produced resembling the vasculature of scaffold structure [199].

- TIPS and porogen leaching

This technique utilizes the well-known porogen leaching technique in which usually water-soluble salt (e.g., NaCl, NaHCO_3_, sugar) is mixed with the polymer and then extracted, leaving pores with predefined size and morphology [200]. Since water is usually used as a sedimenting solvent in TIPS technology, porogen leaching and TIPS can be done with the same substance; in the first step, water is removed in the freeze-drying process and then added again to leach the porogen [201].

- TIPS and textile technology

In this approach, textiles are used because of their excellent mechanical and manufacturing properties. However, big spaces between the threads are often a disadvantage [202]. The strips of the textile are immersed in a polymer solution and quenched to low temperature. Phase separation is then induced by agitation, and the solvent is leached by water or other non-mixing with polymer solvents. The obtained fabrics are uniformly encapsulated and show a highly porous structure [202,203]. They can be sutured to the surrounding tissue and reinforce the mechanical properties [198]. 

## 6. Conclusions and Future Outlooks

The combination of polyurethane and POSS molecules led to the development of important applications, mainly as scaffolds and cardiovascular products, including stents, valves, and membranes, and in the field of bio-imaging and antibacterial coatings. However, most of those applications use only one type of PU matrix: poly(carbonate-urea)urethane, and its derivatives containing various concentrations of polycaprolactone as a biodegradable component of the main backbone. Those matrices are either copolymerized with trans-cyclohexanediolisobutyl-POSS or telomerised with trans-cyclohexanechloroydrinisobutyl POSS. This composition went into further commercial development under the name UCL-NanoBio^™^ and was found to be a good solution in many biomedical applications. Because of that success, the matrix seems to be not subjected to further changes. What is new are the fascinating modification possibilities by chemical and physical methods to achieve better compatibility, porosity, and desired functionalization. For instance, plasma surface treatment and thermally induced phase separation proved efficient modification methods.

Nevertheless, attempts are made to obtain other biocompatible matrices, and some of them appear to have great potential. On the POSS side, numerous research efforts are dedicated to chemical functionalization, including chemical bonding of drug molecules and utilization of self-assembly effects. An inspiring way of further topic exploration may be the incorporation of POSS into NIPU matrices, primarily because of their natural hydrophilicity that does not have to be induced by further modifications.

## Data Availability

Not applicable.
